# Eggplant (*Solanum* spp.) Fruits Dietary Polyphenols Upregulate the Expression of Glucose Transporter Protein in Palmitate-Induced Diabetic Cell Line C2C12

**DOI:** 10.3390/ijms26167762

**Published:** 2025-08-11

**Authors:** Esther E. Nwanna, Emmanuel Mukwevho, Emmanuel Okello, Ademola O. Ayeleso, Emmanuel O. Ibukun, Ganiyu Oboh

**Affiliations:** 1 Department of Biochemistry, Federal University of Technology, P.M.B. 704, Akure 340252, Nigeria; 2 Veterinary Medicine Teaching and Research Center, School of Veterinary Medicine, University of California, Davis, Tulare, CA 93274, USA; 3 Department of Biochemistry, Northwest University, Private Bag X2046, Mmabatho 2735, South Africa; 4 Department of Population Health & Reproduction, School of Veterinary Medicine, University of California, Davis, Davis, CA 95616, USA; 5 Biochemistry Programme, Bowen University, P.M.B. 284, Iwo 220103, Nigeria; ademola.ayeleso@bowen.edu.ng; 6 Department of Life and Consumer Sciences, University of South Africa, Florida Park, Roodeport 1709, South Africa

**Keywords:** rutin, cell line, gene expression, glucose uptake, *GLUT4*

## Abstract

Studies utilizing cell-based systems to investigate plant-based diets for diabetes management are gaining attention due to the adverse effects associated with commercially available drugs. However, the molecular mechanisms underlying the anti-diabetic effects of specific plant-derived products remain inadequately explored. The major aim of our study was to elucidate the molecular mechanisms by which bioactive compounds in the fruit of *Solanum* spp. influence key proteins associated with type 2 diabetes. The expressions of genes such as glucose transporter protein 4 (*GLUT4*), myocyte enhancer factor-2 (*MEF-2A*), and nuclear respiratory factor-1 (*NRF-1*) were investigated in a palmitate-induced C2C12 cell model of type 2 diabetes mellitus. The structures of these proteins were retrieved from the protein database, while bioactive compounds previously identified in *Solanum* spp. were obtained from PubChem site. Drug-likeness properties of these compounds (ligands) were assessed. The docked protein-ligand complexes were further analyzed using the Protein-Ligand Profiler web server. Our results showed that the studied compounds from *Solanum* spp. profoundly upregulated *GLUT4* expression (9–19-fold increase) in the C2C12 cell line, thus surpassing the effects of the standard anti-diabetic drug metformin. Additionally, activities of antioxidant enzymes catalase, superoxide dismutase, and glutathione peroxidase were elevated. Molecular docking showed that rutin, an abundant flavonoid from *Solanum* spp., had the highest binding affinity for the active sites of the target proteins. These findings provide new mechanistic insight into the anti-diabetic effects of *Solanum* spp., primarily due to its high rutin content, which plays a major role in the plant’s glucose-regulating and antioxidant actions. Our findings underscore the potential use of *Solanum* spp. as an affordable functional food for managing type 2 diabetes, especially in developing countries with limited resources for purchasing drugs. Although promising, our findings should be further validated by clinical studies.

## 1. Introduction

Polyphenols have gained significant attention due to their broad biological activities [1], particularly in metabolic disorders such as diabetes mellitus. Found in high concentrations in plant-derived foods, polyphenols possess antioxidant, anti-inflammatory, hepatoprotective, hypolipidemic, antiallergic, and anticancer properties [2,3]. Among these, functional diets rich in polyphenols have been explored as potential therapeutic alternatives to conventional diabetes treatments, aiming to mitigate oxidative stress and improve glucose metabolism [2,3].

Type 2 diabetes mellitus (T2DM) is characterized by prolonged hyperglycemia, which exacerbates oxidative stress [2,3], thus impairing the endogenous antioxidant defense system [3]. This oxidative burden contributes to insulin resistance, beta-cell dysfunction, and associated complications such as neuropathy and cardiovascular disease. Managing hyperglycemia effectively is essential in preventing these complications, necessitating interventions that enhance insulin sensitivity and glucose uptake. Though, it has been reported and confirmed in a review by [3] that medicinal plants products exert anti-diabetic effects through multiple scientifically studied pathways such as insulin secretion, sensitivity, enzyme inhibition, antioxidant activity, anti-inflammatory effects, and in glucose transport such as *GLUT4* activation [3,4]. While conventional pharmaceutical interventions such as metformin, acarbose, etc., depending on the drug class [5], are effective but often associated with side effects which necessitate the use of alternative therapeutic and or strategies from natural products.

Eggplant fruits are known to be rich in bioactive compounds such as phenolic acids, anthocyanins, glycoalkaloids, flavonoids, and saponins that contribute to their pharmacological and nutritional properties [6,7]. However, significant controversies persist regarding the efficacy, bioavailability, and long-term safety of its diets or usage for diabetes management. Many studies rely on in vitro or animal models, limiting direct clinical applicability [8]. Additionally, the variability in dietary bioactive content due to several factors such as methods used in processing, gut microbiota metabolism, and individual genetic differences still raises concerns about reproducibility and effectiveness in diverse populations.

Skeletal muscle plays a crucial role in insulin-mediated glucose disposal, as it accounts for the majority of postprandial glucose uptake [4]. The translocation of glucose transporter 4 (*GLUT4*) from intracellular compartments to the plasma membrane is a critical mechanism for glucose homeostasis. The process is mediated through the insulin signaling pathway, primarily involving the activation of myocyte enhancer factor-2 (*MEF-2A*) and nuclear respiratory factor-1 (*NRF-1*), which regulate *GLUT4* expression [8]. Disturbances hitting these routes are a hallmark of insulin resistance, highlighting the necessity for therapeutic interventions targeting *MEF-2A* and *NRF-1*, which was confirmed by Mukwevho’s study [4].

We have previously studied the effects of diet supplemented with eggplant (*Solanum* spp.) on streptozotocin-induced T2DM in a Wistar rat model [1]. In continuation of our research, we further investigated the anti-diabetic potential of the same dietary regime supplemented with eggplant (*Solanum* spp.), but we employed a palmitate-induced C2C12 cell model designed for exploring T2DM at the molecular level by evaluating the expression of *GLUT4*, *MEF-2A*, and *NRF-1*, alongside activities of intracellular antioxidant enzymes such as glutathione peroxidase (GPx), catalase, and superoxide dismutase (SOD), we aimed to elucidate the molecular mechanisms underlying the observed glucose-lowering effects. Moreover, by using molecular docking, we explored the interactions between key bioactive compounds from the eggplant and *GLUT4*, thus providing mechanistic insights into their therapeutic potential. Given the urgent need for cheap, safe, and effective diabetes interventions, this study seeks to validate the role of *Solanum* spp. as a functional dietary strategy with minimal or no adverse effects, contributing to the ongoing debate surrounding plant-based diabetes management approaches.

## 2. Results

**Figure 1:**
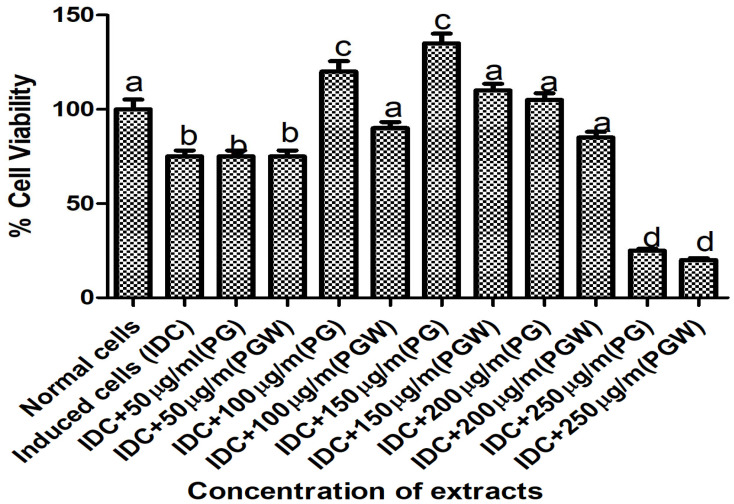
Cell toxicity assay using MTT at different concentrations of eggplant extract. Bars with the same letter are not significantly different (*p* < 0.05). **KEY**: PG: *Solanum kumba* PGW: *Solanum aethiopicum.* IDC means induced cells which means normal cells with MTT solution.

The cell viability and extract toxicity assay were conducted on the C2C12 cell line to determine the optimal, non-toxic concentration of the eggplant extract for the experiment. MTT measures the cell viability (percentage of live cells/total number of cells). We employed the MTT assay to measure the viability of C2C12 cells incubated with increasing concentrations of the eggplant extract: [50, 100, 150, 200, and 250] µg/mL The results indicated that 150 µg/mL was the highest concentration at which cellular activity and viability remained comparable to the positive control (normal cells), making it the selected concentration for our subsequent experiments (Figure 1).

**Figure 2:**
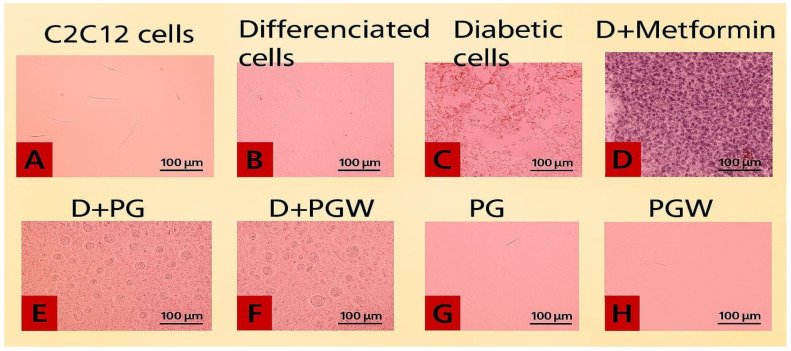
Shows the image of the cell population of the C2C12 cell plates at Magnification: 100×; Scale Bar: 100 μm. **KEY**: (**A**): C2C12: Normal cells without the myotubes; (**B**): Differentiated cells Positive Control (healthy matured cells) with myotubes; (**C**): Negative control induced with 0.75 mM Palmitate (diabetic cells); (**D**): Induced with 0.75 mM Palmitate + 0.1 µM Metformin (diabetic cells treated with metformin); (**E**): Induced with 0.75 mM Palmitate + PG (diabetic cells treated with 150 µg/mL *Solanum kumba*); (**F**): Induced with 0.75 mM Palmitate + PGW (diabetic cells treated with 150 µg/mL *Solanum aethiopicum*); (**G**): Healthy matured cells with 150 µg/mL PG (*Solanum kumba*); (**H**): Healthy matured cells with 150 µg/mL PGW (*Solanum aethiopicum*).

The images of the C2C12 cell plates, as presented in Figure 2, illustrate the effects of different treatments on cultured C2C12 cells. The cells were initially maintained in Dulbecco’s Modified Eagle’s Medium (DMEM) supplemented with 10% fetal calf serum (FCS) at 37 °C in a humidified atmosphere containing 5% CO_2_ for three days, allowing them to reach 80–90% confluence (Figure 2A). To facilitate myocytic differentiation, the 10% FCS was then replaced with 2% FCS for an additional two days (Figure 2B). The differentiated mature cells were subsequently used for the diabetes experiment (Figure 2C–H).

In the experimental setup, Figure 2B served as the positive control, containing healthy, mature cells with normal growth media. Figure 2C contained untreated diabetic cells, serving as the negative control. Figure 2D–F consisted of type 2 diabetic cells subjected to eggplant extract treatment: Figure 2D was treated with 0.1 µM metformin, Figure 2E with 150 µg/mL *Solanum Kumba*, and Figure 2F with 150 µg/mL *Solanum aethiopicum*. Additionally, Figure 2G,H contain healthy mature cells supplemented with 150 µg/mL *Solanum kumba* (PG) and *Solanum aethiopicum* (PGW), respectively.

The results indicated structural changes and variations in cell population among the groups. The untreated diabetic cells (Figure 2C) exhibited a notable reduction in cell density, with visible empty spaces indicating cell death. In contrast, the metformin-treated group (Figure 2D) and the eggplant extract-treated groups (Figure 2E,F) demonstrated enhanced cell recovery, suggesting that both metformin and eggplant extract diets effectively mitigated the palmitate-induced damage, leading to a restoration of cell confluence, although structural differences were observed from the image.

**Figure 3:**
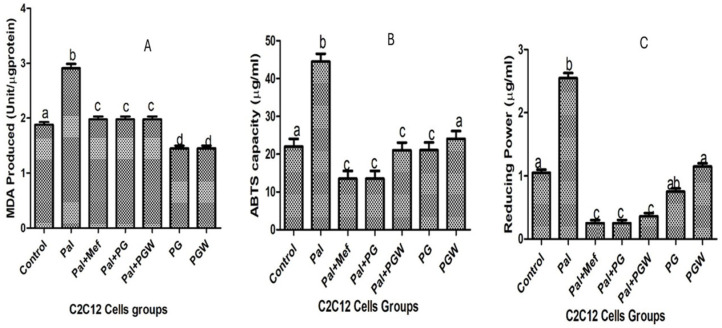
Intracellular antioxidant activities and lipid peroxidation of the eggplant extracts on MDA (**A**), produced ABTS (**B**), and FRAP (**C**). Bars with the same letter are not significantly different (*p* < 0.05). **KEY:** from the left to the right on the bar chart (Control): is normal cells (Pal): is the diabetic cells without treatment, (Pal + Mef): is diabetic cells with metformin, (Pal + PG): diabetic cells + *Solanum kumba*, (Pal + PGW): diabetic cells + *Solanum aethiopicum*, (PG): normal cells + *Solanum kumba*, (PGW): normal cells + *Solanum aethiopicum*.

**Figure 4:**
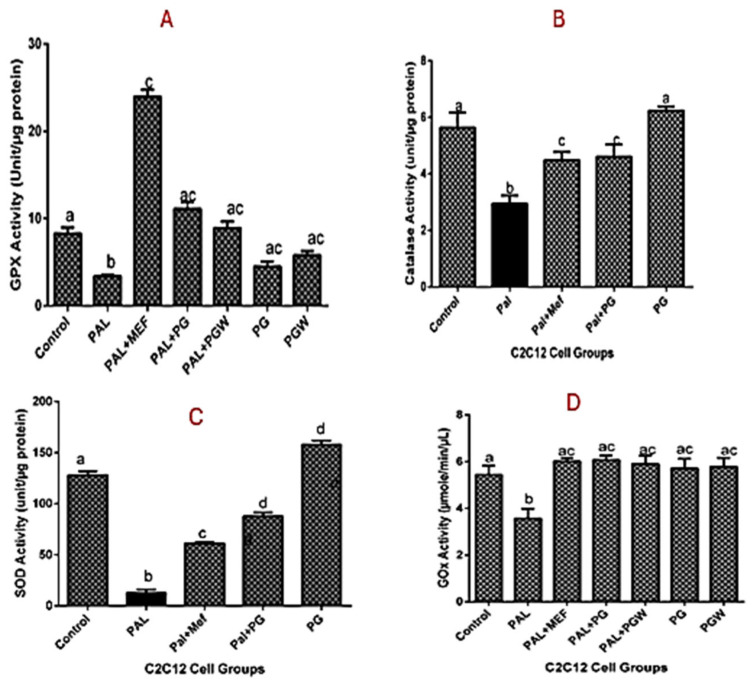
Intracellular effect of eggplant extracts on the antioxidant enzymes glutathione peroxidase (**A**), catalase (**B**), superoxide dismutase (**C**), and glucose oxidase (**D**) activities in C2C12 cells. Values represent mean ± standard deviation (n = 3). Bars with the same letter are not significant (*p* < 0.05). **KEY:** from the left to the right on the bar chart (Control): is normal cells (Pal): is the diabetic cells without treatment, (Pal + Mef): is diabetic cells with metformin, (Pal + PG): diabetic cells + *Solanum kumba*, (Pal + PGW): diabetic cells + *Solanum aethiopicum*, (PG): normal cells + *Solanum kumba*, (PGW): normal cells + *Solanum aethiopicum*.

The lipid peroxidation in C2C12 cells originating from oxidative stress can be inhibited by cell treatment with eggplant extracts. The extent of lipid peroxidation is directly proportional to the production of MDA, the product of lipid peroxidation (Figure 3A). The production of MDA is increased in C2C12 cells pre-exposed to palmitate but reversed to normal levels in cells that were subsequently treated with PG or PGW. The positive effects of eggplant extracts are comparable to those of the anti-diabetic drug metformin (Figure 3A). The effect of eggplant extracts on the lipid peroxidation vis a vis total antioxidant property (ABTS) and ferric reducing antioxidant properties (FRAP) on C2C12 cells were presented in Figure 3B,C, treatment with metformin and the eggplant supplemented diet restored the antioxidant status of the cells significantly (*p* < 0.05) although there was no significant change (*p* > 0.05) in the healthy cell groups and the controls. The effect of the antioxidative property of the eggplant diet on intracellular enzymes, superoxide dismutase (SOD), catalase, and glutathione peroxidase (GPX) (Figure 4A–C) was observed, which followed the same trend as the positive control group. Although the enzyme activities of the cells treated with eggplant extracts were better when compared with the metformin-treated group.

Glucose uptake (GOX) activity was assessed to determine insulin sensitivity and resistance among the experimental groups, as presented in Figure 4D. The results demonstrated an increase in the glucose uptake across all treatment groups. However, the untreated diabetic group exhibited the lowest GOX activity compared to the groups treated with metformin and eggplant extract, as well as the control group. These findings suggest that both metformin and the eggplant-supplemented diet effectively enhanced glucose uptake, mitigating the insulin resistance observed in the untreated diabetic cells.

**Figure 5:**
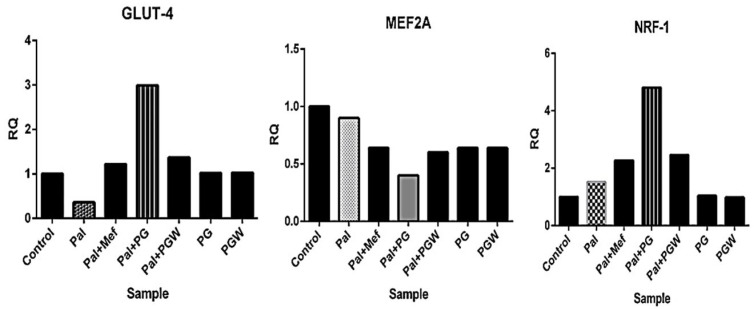
The genetic expression with RT-qPCR on *GLUT4*, *NRF-1*, and *MEF-2A* in C2C12 cells.

The expression of genes encoding *GLUT4*, *MEF-2A*, and *NRF-1*, the relative abundance of the gene transcripts was measured using Real-Time Quantitative Polymerase Chain Reaction (RT-qPCR), which was performed in triplicate using the Step One Plus TM PCR machine (Applied Biosystems, Foster City, CA, USA) with the PowerUp SYBR Green master mix. Specific primers were utilized to amplify target regions corresponding to the *GLUT4*, *MEF-2A*, and *NRF-1* genes, which are associated with T2DM (Table 1). The thermal cycling conditions consisted of an initial holding cycle at 50 °C for 2 min, followed by a second holding cycle at 95 °C for 2 min. This was followed by 40 amplification cycles, with denaturation at 95 °C for 3 s and annealing/extension at 60 °C for 30 s. Negative control, devoid of a cDNA template, was included in each of the assays to assess background signal and ensure result reliability. The relative mRNA concentrations were determined based on changes in threshold cycle (Ct) values. The presence of these mRNAs was visible in the experimental samples, confirming their presence and expression across all treatment groups, as evidenced by the amplification plots (available upon request). The expression level is presented in Figure 5, while the fold change is detailed in Table 2. The results indicate a significant upregulation of gene expression (*NRF-1* and *GLUT4*) in the treatment groups receiving metformin and eggplant extract, while *MEF-2* was downregulated.

Relative amplification relative to housekeeping gene is 1 as seen in the table for the positive control group A for all the expressed genes, *NRF-1* expression must increase to facilitate the binding site for *MEF-2A* while *MEF-2A* decrease in order to promote transcription for *GLUT4* expression this was observed for groups B to G, notably, the diabetic cells supplemented with *Solanum kumba* (PG) group D exhibited the highest *GLUT4* expression, with a 2.99-fold increase relative to positive and negative control groups (A&B). This was followed by *Solanum aethiopicum* group E, which also demonstrated greater translocation and upregulation of *GLUT4* with the eggplant-supplemented diet when compared to metformin (1.22-fold). These findings suggest that the rutin-rich *Solanum* spp. diet exerts a more pronounced effect on *GLUT4* expression and better glucose uptake than the conventional anti-diabetes drug used in the T2DM model. The observed *NRF-1* upregulation suggests that *Solanum* spp. improves glucose metabolism by enhancing *GLUT4* transcription via the *NRF-1* to *MEF-2A* pathway (Figure 5). In addition, the fold change (Table 2) shows the relative quantification (RQ), but when RQ = 1, it means there is no change in gene expression, but when it is more than 1 (RQ > 1), it means there is gene upregulation and expression. This means that the treatment of *Solanum* spp. extract diet on the palmitate-induced C2C12 cells and metformin increases the genetic expression of our protein of interest *GLUT4*, which has an RQ value of 2.99, meaning the *GLUT4* gene expression is 2.99 times higher in the treated sample compared to the negative control. *GLUT4* expression supports the hypothesis that *Solanum* spp. enhances glucose metabolism.

**Figure 6:**
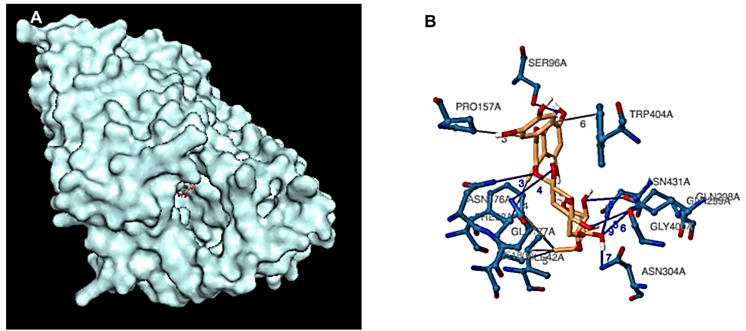
(**A**) Binding site for Rutin within, (**B**) Molecular interaction of rutin with amino acid residues within the binding pocket of *GLUT4*.

**Figure 7:**
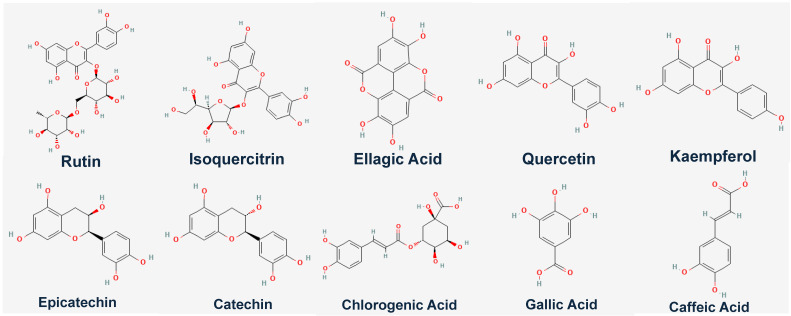
Structures of compounds source from PUBCHEM site: https://pubchem.ncbi.nlm.nih.gov/ (accessed on 2 April 2024).

To further investigate how the individual bioactive compounds work computationally aside from the genetic expression of *GLUT4* in C2C12 cell line, we observed that ten compounds/ligands identified from our previous studies [1,2] were docked individually with our protein of interest (*GLUT4*, *MEF-2A*, and *NRF-1*) using different software, however, it was observed that rutin had the highest binding affinity. Table 3 shows the binding energies (kcal/mol) of eggplant compounds docked against *GLUT4*, *MEF-2A*, and *NRF-1* using the Protein-Ligand Profiler web server only the molecular interaction of rutin with amino acid residues within the binding pocket of *GLUT4* was shown in (Figure 6). Compounds/Ligands structures (Figure 7) and CID numbers (Table 3) were retrieved from the PUBCHEM site, and protein IDs were retrieved from the protein data bank site (PDB) according to the methods of [8,9,10].

## 3. Discussion

This study evaluates *Solanum* spp. bioactive compounds, specifically rutin, for diabetes management using in vitro and in silico methods. Results show that rutin might have increased *GLUT4* expression and antioxidant defenses in C2C12 cells, suggesting improved insulin sensitivity and glucose uptake. In silico docking analysis demonstrates high binding affinity of rutin to *GLUT4*, *MEF-2A*, and *NRF-1*, indicating potential for glucose regulation, mitochondrial function, and oxidative stress reduction. These findings support plant-based therapies as viable options for type 2 diabetes with minimal adverse effects. This study reinforces the growing body of evidence supporting the role of plant-based bioactive compounds in diabetes management [11,12]. The significant upregulation of *GLUT4* expression (9–19-fold increase) in palmitate-induced C2C12 diabetic cell line treated with rutin-rich *Solanum* spp. highlights a promising dietary intervention. This result surpasses the effects of metformin, a widely used anti-diabetic drug suggesting that specific plant-derived compounds can influence key metabolic pathways [2,3]. The enhanced *GLUT4* expression suggests that rutin and other bioactive compounds in *Solanum* spp. have antioxidative and anti-diabetes properties which could have facilitate glucose uptake by modulating insulin-independent pathways. Previous studies [13,14] indicate that *MEF-2A* plays a crucial role in *GLUT4* translocation, particularly in response to non-insulin stimuli such as exercise and dietary polyphenols. Similarly, *NRF-1* has been identified as a key regulator of mitochondrial biogenesis and glucose metabolism, and its upregulation in response to *Solanum* spp. suggests a broader metabolic benefit beyond glucose metabolism [14,15]. The decrease in *NRF-1* in healthy cells suggests that under normal physiological conditions, *NRF-1* is maintained at lower level because there is no metabolic stress requiring mitochondrial compensation. In diabetic cells, *NRF-1* is upregulated as an adaptive response to counteract oxidative stress and impaired mitochondrial function. This aligns with our study findings that *Solanum* spp. upregulates *NRF-1* expression potentially enhancing mitochondrial activity and glucose metabolism in diabetic conditions as reported [16,17,18,19]. For example, flavonoid-rich diets have been shown to ameliorate insulin resistance by modulating oxidative stress and inflammation [16,19]. The study further refines our understanding that rutin [20,21] known for its strong antioxidant property might be a key contributor of effective glucose-lowering agent as seen with its high binding affinity to *GLUT4*, *MEF-2A*, and *NRF-1* through in silico docking analysis. Additionally, metabolic benefits of the eggplant fruits diet are demonstrated through increased SOD, catalase, and GPX activities which support the notion that reducing oxidative stress is a viable strategy in diabetes management [16]. Despite these promising findings, several limitations that must be addressed in future research such as clinical trials are necessary to validate the efficacy of *Solanum* spp. diet in diabetic patients. Additionally, the bioavailability and metabolism of eggplant diet supplementation need further investigation to determine optimal intake and potential interactions with conventional anti-diabetic medications. Other compounds activities were not fully ruled out while rutin was identified as the most active compound based on molecular docking. This study did not conduct experiments with isolated rutin to definitively confirm that its activity alone is responsible for the observed effects, and the potential interactions of rutin and other bioactive compounds in *Solanum* spp. were not investigated.

## 4. Materials and Methods

### 4.1. Sample Collection and Preparation

Matured fruits of eggplant *Solanum* varieties (*aethiopicum* and *kumba*) were obtained from Erekesa market, Akure in Ondo state, Nigeria. The person responsible for sample identification was Mr. Omomoh, affiliated with the Department of Forestry and Wood Technology, Federal University of Technology, Akure, Nigeria. The herbarium numbers assigned were IFE-17718 for *Solanum kumba* and IFE-17719 for *Solanum aethiopicum*, with the samples deposited at the FUTA Centre for Research and Development (CERAD) Herbarium. Standard fruit processing techniques were employed, including cleaning, sorting, cutting, pulping, drying, and grinding, following established methodologies [22]. Here, 5 g of dried, pulverized eggplant material was extracted with 50 mL of distilled water by continuous agitation on an orbital shaker for 4 h at room temperature. The resulting mixture was subsequently filtered using Whatman No. 1 filter paper (Cytiva, Kent, UK) to remove particulate matter. The aqueous filtrate was then lyophilized (freeze-dried) with (FreeZone 2.5 Liter Benchtop Freeze Dry System Labconco Corporation, Kansas City, MO, USA) to obtain a dry extract yielding a final solute concentration of approximately 5 µg, which was stored in −4 °C used for subsequent analyses.

### 4.2. Chemicals and Reagents

PureLink RNA Mini Kit from Thermo Fisher Scientific (Invitrogen), Burlington, MA, USA, Palmitic acid (C18:0) from Chemie GmbH, (Steinheim am Albuch, Germany), Bovine serum albumin BSA (catalog number A4919), metformin (Sigma-Aldrich (MilliporeSigma) from St. Louis, MO, USA) Glucose Oxidase Activity Assay Kit was obtained from (Invitrogen Thermofisher, Waltham, MA, USA). Bradford protein assay kit was obtained from Bio-Rad Laboratories (Hercules, CA, USA). Protease tablets were purchased from Roche (South San Francisco, CA, USA), while reverse and forward primers were gotten from Integrated DNA Technologies (Coralville, IA, USA) as well as the reference gene β-actin, Murine C2C12 skeletal muscle cells (CRL 1722) were obtained from the American Type Culture Collection (Manassas, VA, USA) and cell culture kits for cellular antioxidants were supplied by Sigma Aldrich (St. Louis, MO, USA). The water was glass-distilled, and all other chemicals and reagents were of analytical grade.

### 4.3. Cytotoxicity Assay

Determination of mitochondrial activity was performed using (3-(4,5-dimethylthiazol-2-yl)-2,5-diphenyltetrazolium bromide) (MTT). The MTT assay is a colorimetric method for assessing cell metabolic activity. It is used to evaluate cell viability and proliferation [23]. C2C12 cells derived from mouse muscle were sub-cultured and differentiated, then seeded into 24-well plates (25,000 cells/well), followed by the addition of eggplant extracts at different concentrations ranging from 50 µg/mL to 250 µg/mL. Cells were incubated for 24 h in 100% Dulbecco’s Modified Eagle medium (DMEM) while other processes were carried out as described by [23]. The absorbance at 570 nm was read using a plate reader and subsequently calculated. Concentrations of the extracts that decrease the cell count when compared to the control cell were calculated as:

$$\mathrm{MTT}\;\mathrm{cytotoxicity}\;(\%)\;=\;\frac{Absorbance\;of\;sample}{Absorbance\;of\;control}\;\times \;100$$

### 4.4. Treatment of Cells with Palmitate

Palmitate-containing culture media were prepared using a method described by [14] with slight modifications. Briefly, palmitate was dissolved in heated ethanol (95 °C) to a final concentration of 75 mM, filter-sterilized, and diluted (1:100) in DMEM containing 2% BSA to yield a final palmitate concentration of 0.75 mM. The palmitate solution in the BSA + DMEM was left at 37 °C for 60 min to allow palmitate to conjugate with BSA. Except for the control cells plate, which has no palmitate +BSA and only DMEM, the other plates containing 0.75 mM palmitate solution, which form adducts with BSA in DMEM, were added to the plates using standard protocol, and all plates were left for 16 h. Thereafter, cells were glucose- and serum-starved. The previous DMEM solution was decanted and then incubated in the plates with phosphate buffer saline (PBS) at 37 °C in 5% CO_2_ and humidified air for 30 min. After starvation, the cells were exposed to DMEM, which contained 8 mM glucose in all the plates, while treatment with eggplant extracts (150 µg/mL), and 0.1µM metformin (positive control) were added and left for 3 h at 37 °C in 5% CO_2_ humidified air. There were seven groups: group one (control healthy cells), group two (Negative control that is diabetic), group three (diabetic and treated with 0.1 µM metformin), group four (diabetic and treated with 150 µg/mL *Solanum kumba* (PG)), group five (diabetic and treated with 150 µg/mL *Solanum aethiopicum* PGW), group six (healthy cells with 150 µg/mL *Solanum kumba* (PG)), and group seven (healthy cells with 150 µg/mL *Solanum aethiopicum* PGW). Thereafter, the culture-treated C2C12 cells were washed with phosphate buffer (pH 7.4). The washed cells were detached from each plate surface with a cell scraper into 2 mL Eppendorf tubes and were cold centrifuged at 2000× *g*. The residue was dissolved in 1 mL phosphate buffer (pH 7.4). Afterward, the cells were sonicated, and the cell lysates were used for the subsequent assays.

### 4.5. Cellular Antioxidant Assay

Trolox equivalent antioxidative capacity (TEAC) analysis of C2C12 cells was carried out using the protocol described in [24] and modified, and ferric reducing antioxidant property (FRAP) was performed as described by [25]. The lipid peroxidation assay measuring malondialdehydes (MDA) and 2-azinobis (3-ethylbenzothiazoline-6-sulfonate) radical (ABTS+) was carried out using the modified method of [26]. Proteins in C2C12 cells were measured by the Bradford assay kit. The enzymatic activities of glutathione peroxidase (GPx) and catalase were measured as described by [27,28]. While the Sigma Aldrich kit was used for superoxide dismutase, insulin sensitivity was measured using a glucose oxidase (GOx, EC 1.1.3.4) kit from Invitrogen Thermofisher USA.

### 4.6. Bioassay on Genetic Expression

From the C2C12 cell-line experimental groups, RNA was extracted and purified, following the kit from the Sigma Aldrich manufacturer’s instructions. The purified RNA was stored at −80 °C until used. RNA was used for cDNA synthesis and subsequently for genetic expression. Nanodrop was used to determine the quality and the concentration of the RNA yield.

### 4.7. Determination of RNA Integrity Using Gel Electrophoresis

Agarose gel was prepared with Tris-base acetic acid (TAE) buffer mixed with Ethylenediaminetetraacetic acid (EDTA). The mixture was heated in the microwave for 60 s. It was allowed to cool to about 50 °C, 1.5 µL of ethidium bromide was added, thoroughly mixed, and the gel was poured into a gel tray and allowed to solidify. A mixed portion (7 µL of the RNA extracted from the previous experimental groups) and 3 µL of the loading dye) were mixed and loaded into the wells for each of the experimental groups, and this was allowed to run for 80 min at 60 volts. After the run time, the gel was viewed with EVOS Imaging System for multiplexed tissue and cell imaging, Thermo Fisher Scientific.

### 4.8. Quantitative Polymerase Chain Reaction (qPCR)

The qPCR was performed in triplicate using the Step One Plus TM PCR machine (Applied Biosystems, Foster City, CA, USA) was used to synthesize double-stranded cDNA. 2 µg of total RNA were used to amplify the region of the following genes (*GLUT4*, *MEF-2A*, and *NRF-1*) alongside the housekeeping gene or reference gene β-Actin. Relative mRNA expression used β-Actin reference gene to normalize the synthesis.

### 4.9. In-Silico Study

Structures of the selected polyphenol compounds were retrieved from the PubMed Chem database (https://pubchem.ncbi.nlm.nih.gov/, accessed on 2 April 2024) in SDF format while the three-dimensional crystal structures of the proteins were accessed from the Protein Database (PDB) (https://www.rcsb.org/, accessed on 2 April 2024) the protein PDB ID for homo sapiens *GLUT4* profiler (7WSM) Yuan et al., 2022 [8], *MEF-2A* profiler (3MU6) Jayathilaka et al., 2012 [9] and NRF-1 profiler (4L7B) Jnoff et al., 2014 [10] were retrieved.

The three crystal structures were first subjected to dock preparation using tool-UCSF-Chimera© software, version 1.13 (http://www.cgl.ucsf.edu/chimera, accessed on 2 April 2024), followed by minimization and subsequent molecular docking, which was conducted through a flexible docking procedure employing PyRx 0.8, a suite integrated with Auto Dock Vina. The specific target sites for the receptors corresponding to the substrate-binding regions were adjusted using the grid box with dimensions. The center was attuned based on the site of substrate binding in the respective protein (Table 3), thus revealing compounds with the best binding score at the end of the experiment.

### 4.10. Data Analysis

The mean average value of the replicates was calculated. Tukey test and one-way analysis of variance with GraphPad Prism version 7.0 were used to analyze the results, and Duncan multiple tests were used for the post hoc [29]. A significant difference was taken to be (*p* < 0.05).

## 5. Conclusions

This study provides compelling evidence that *Solanum* spp. rich in bioactive compounds can significantly enhance glucose metabolism and increase antioxidant defense mechanisms. These findings support the use of plant-based interventions for metabolic health, especially diabetes mellitus. As the search for effective T2DM treatments continues, functional foods like *Solanum* spp. offer a promising avenue for future research and therapeutic development.

### Recommendation

Future research will focus on experiments using purified rutin and exploring its potential synergistic interactions with other polyphenols present in eggplant fruits.

## Figures and Tables

**Table 1:** Forward, reverse, and reference genes used for PCR.

Genes	Forward Primer	Reverse Primer
*GLUT4*	5′-GCA GCG AGT GAC TGG AAC A-3′	5′-CCA GCC ACG TTG CAT TGT AG-3′
*NRF-1*	5′-AAA CAC AAA CTC AGG CCA CC-3′	5′-CCA TCA GCC ACA GCA GAG CA-3′
*MEF-2A*	5′-GTG TAC TCA GCA ATG CCG AC-3′	5′-AAC CCT GAG ATA ACT GCC CTC-3′
*β-Actin*	5′-GAG ACC TTC AAC ACC CCA GCC-3′	5′-GGA GAG CAT AGC CCT CGT AG-3′

**Table 2:** Fold change of genetic expression in palmitate-induced C2C12 in RT-qPCR.

Groups	*GLUT4*	*NRF-1*	*MEF-2A*
A	1	1	1
B	0.16	1.52	0.90
C	1.22	2.27	0.64
D	2.99	4.81	0.40
E	1.37	2.45	0.60
F	1.02	1.04	0.64
G	1.03	1.01	0.64

**KEY**: A: Positive control (normal cells). B: Negative control (diabetic cells without treatment). C: diabetic cells + Metformin. D: diabetic cells + PG (*Solanum kumba*). E: diabetic cells + PGW (*Solanum aethiopicum*). F: normal cells + PG (*Solanum Kumba*). G. normal cells + PGW (*Solanum aethiopicum*).

**Table 3:** In silico prediction of binding sites for Rutin located on *GLUT4*, *MEF-2A* and *NRF-1* using Protein-Ligand Profiler web server.

CID	Compounds	Docking Score *GLUT4*	CID	Compounds	Docking Score *MEF-2A*	CID	Compounds	Docking Score *NRF-1*
5280805	Rutin	−12.999	5280805	Rutin	−6.102	5280805	Rutin	−12.734
5280804	Isoquercitrin	−10.72	5280804	Isoquercitrin	−3.483	5281855	Ellagic acid	−11.125
5281855	Ellagic acid	−10.102	72276	Epicatechin	−3.476	5280804	Isoquercitrin	−10.961
5280343	Quercetin	−9.018	1794427	Chlorogenic acid	−3.121	9064	Catechin	−10.665
5280863	Kaempferol	−8.995	689043	Caffeic acid	−2.781	1794427	Chlorogenic acid	−9.78
72276	Epicatechin	−8.78	5280863	Kaempferol	−2.494	370	Gallic acid	−8.581
9064	Catechin	−8.74	370	Gallic acid	−2.112	5280343	Quercetin	−8.556
1794427	Chlorogenic acid	−7.317	9064	Catechin	−1.314	5280863	Kaempferol	−7.346
370	Gallic acid	−6.964	5280343	Quercetin	−0.184	72276	Epicatechin	−6.466
689043	Caffeic acid	−5.82	370	Gallic acid	0.961	689043	Caffeic acid	−5.795

## Data Availability

The datasets used and/or analyzed during the current study are available from the corresponding author on reasonable request.
